# Neonatal Outcomes of Infants Diagnosed with Fetal Growth Restriction during Late Pregnancy versus after Birth

**DOI:** 10.3390/jcm13133753

**Published:** 2024-06-27

**Authors:** Ohad Houri, Meytal Schwartz Yoskovitz, Asnat Walfisch, Anat Pardo, Yossi Geron, Eran Hadar, Ron Bardin

**Affiliations:** 1 Helen Schneider Hospital for Women, Rabin Medical Center, Petach Tikva 4941492, Israel; meytalsch7@gmail.com (M.S.Y.); asnatwa@clalit.org.il (A.W.); pardoanat@gmail.com (A.P.); yosgeron@gmail.com (Y.G.); eranh42@gmail.com (E.H.); ronbardin@gmail.com (R.B.); 2 Faculty of Medicine and Health Science, Tel Aviv University, Tel Aviv 6997801, Israel

**Keywords:** fetal growth restriction, adverse neonatal outcomes

## Abstract

**Objective:** The aim of this study was to investigate the potential differences in the outcomes of neonates in whom FGR was diagnosed late in pregnancy as compared to those in whom growth restriction was diagnosed after birth. **Methods:** A retrospective study was conducted in a tertiary medical center between 2017 and 2019. The study included women carrying a single infant with an estimated fetal weight below the tenth percentile in whom FGR was diagnosed during late pregnancy, after 32 gestational weeks (known late-onset FGR; study group) or only after birth (unknown FGR; control group). Data were collected by review of the electronic health records. The primary outcome measure was the rate of composite adverse neonatal outcome. **Results:** A total of 328 women were included, 77 (23.47%) in the known-FGR group and 251 (75.53%) in the unknown-FGR group. In the known-FGR group, an etiology for the FGR was identified in 28.57% cases, most commonly placental insufficiency (21.74%). Compared to the unknown-FGR group, the known-FGR group was characterized by significantly higher rates of elective cesarean delivery (15.58% vs. 9.96%, *p* < 0.001), preterm birth (18.18% vs. 3.98%, *p* < 0.01), and labor induction (67.53% vs. 21.51%, *p* < 0.01). A significantly higher proportion of neonates in the known-FGR group had a positive composite adverse outcome (38.96% vs. 15.53%, *p* < 0.01). For multivariate regression analysis adjusted for maternal age, gestational age at delivery, and mode of delivery, there was no difference between groups in the primary outcome (aOR 1.73, CI 0.89–3.35, *p* = 0.1). Every additional gestational week at delivery was a protective factor (aOR = 0.7, 95% CI 0.56–0.86, *p* < 0.01). **Conclusions:** A prenatal diagnosis of late-onset FGR is associated with higher intervention and preterm birth rates as compared to a diagnosis made after birth. Fetuses diagnosed with late-onset FGR during pregnancy should undergo specific and personalized assessment to determine the cause and severity of the growth delay and the best management strategy. This study highlights the importance of careful decision-making regarding the induction of labor in late-onset FGR.

## 1. Background

Fetal growth restriction (FGR) is a condition in which the fetus has not reached its full growth potential. It is defined as an estimated fetal weight (EFW) below the 10th percentile for gestational age [1,2]. FGR is divided into early and late onset based on the time of diagnosis: before (20–30%) and after (70–80%) 32 gestational weeks (GW). There are differences in pathogenesis, severity, course, and outcomes between the two types [3,4,5].

In cases of FGR, delivery is timed to achieve maximum fetal growth and maturity while minimizing short- and long-term morbidity and mortality [6]. There is currently no consensus on the optimal timing of delivery [2,7].

Numerous studies have investigated the timing and effectiveness of prenatal diagnosis, monitoring, and treatment of FGR to improve maternal and neonatal outcomes [7,8,9,10,11,12,13], but the findings are unclear [2,7]. Some showed that diagnosing FGR during pregnancy improved immediate neonatal outcomes and reduced intrauterine mortality [8,9,10], whereas others suggested that diagnosing FGR prenatally increases maternal and neonatal complications [11,12,13], while no significant positive impact on overall mortality is apparent [14]. The aim of this study was to investigate differences in neonatal outcomes between late-FGR neonates who were diagnosed during pregnancy or only after delivery.

## 2. Methods

### 2.1. Design and Setting

A retrospective study was conducted between January 2017 and September 2019 at a single tertiary medical center.

### 2.2. Study Population

The study included women with singleton gestations and a normal pregnancy course (including nuchal translucency, routine fetal anomaly scans at 18–22 weeks gestation, and a glucose challenge test) who gave birth to infants with a birthweight below the 10th percentile for gestational age according to the national growth charts [15]. Participants were classified into two groups: late-onset FGR diagnosed during pregnancy based on sonographic EFW after 32 gestational weeks (known FGR; study group), and FGR first diagnosed at birth according to the actual birthweight (Unknown FGR; comparison group).

In this study, we included only women who had completed recommended fetal investigations before 32 GW with normal results in all antenatal follow up tests. The tests included nuchal translucency, first- and second-trimester biochemical markers, routine anatomical scans, and EFW prior to 32 gestational weeks.

In the known-FGR group, we included only women who have been followed at our high-risk clinic and ultrasound unit.

Exclusion criteria were any anatomical or genetic fetal malformations known before 32 GW, chronic maternal disease (pre-pregnancy diabetes, chronic hypertension, hypo/hyperthyroidism, asthma, epilepsy, lupus, APLA syndrome), known early-onset FGR (before 32 GWs), multiple gestation, missing information on pregnancy follow-up or outcome.

The known-FGR group was further subdivided by etiology of FGR (identified av not identified) during pregnancy.

### 2.3. Definitions and Practice Guidelines

Late-onset FGR was defined as a sonographic EFW below the 10th percentile for gestational age (SMFM, 2020) according to the nationally accepted growth curves representing the norm in the Israeli population, that was diagnosed for the first time after 32 gestational weeks [1,2,7,15].

Uteroplacental insufficiency was defined as a pulsatility index (PI) above the 95th percentile for gestational age in the umbilical artery (UA) or a PI below the fifth percentile for gestational age in the middle cerebral artery (MCA) [2].

Gestational age was calculated according to the date of the last menstrual period and was confirmed by a first-trimester measurement of the crown-rump length. Gestational age was changed if the gap was more than 4 days before 10 gestational weeks [1].

In our health system, an EFW scan is routinely done at 32 GWs. The scan is fully covered by public health insurance. When the EFW is at or below the 10th percentile, Doppler indices are sought as part of the immediate evaluation. This is followed by a comprehensive maternal-fetal evaluation of the medical and maternal history and a thorough physical examination, including weight, height, and weight gain during pregnancy as well as blood pressure measurement to rule out pregnancy-related hypertensive morbidity. According to the Israeli guidelines, when fetal causes are suspected (an EFW less than the third percentile, polyhydramnios, an abnormal anatomical scan, personal family history of genetic disease), a more detailed investigation is offered which is also fully covered by public health insurance [1]. It consists of genetic counseling, late amniocentesis for evaluation of chromosome microarray analysis (CMA), with optional further genetic investigation by whole-exome sequencing (WES), late repeated anatomical scan, fetal echocardiogram, and laboratory tests for viral infections (CMV, toxoplasma). These tests are usually offered and are fully covered even in the absence of additional risk factors for fetal abnormalities except for the EFW.

When late FGR is diagnosed, women are followed at designated maternal–fetal medicine clinics. The EFW is assessed every 2 weeks, and a non-stress test (NST) and biophysical profile (BPP) along with a UA and MCA Doppler assessment are performed weekly. The timing and mode of delivery are based on these findings as well as on gestational age and pattern of fetal growth. Cesarean delivery (CD) is performed for standard obstetric indications. In cases of absent or reverse UA diastolic flow, delivery is recommended at 34 or 32 gestational weeks, respectively. At 37 gestational weeks, if the EFW is lower than the third percentile or Doppler indices are abnormal, oligohydramnios or lack of growth is detected, or there are other physician/maternal concerns, induction of labor is recommended. At the time of this study, the common practice at our center was to deliver any fetus with an EFW or AC below the 10th percentile at 37 GWs, even if there were no other risk factors.

### 2.4. Data Collection

Data were collected by review of the computerized medical records. The hospital’s healthcare databases are valid and highly reliable, including diagnoses based on codes and fixed texts as well as fields for categorical and continuous variables. In cases of uncertainty and/or missing data, a manual reading/scan of the patient’s file was performed.

Maternal, fetal, obstetric, and neonatal parameters were collected. Maternal parameters included age, height, and weight (pre-pregnancy and at delivery) and medical and obstetric history. Fetal parameters included gestational week (GW), EFW, and fetal biometry at the time of the FGR diagnosis. pregnancy follow-up data of up to 32 gestational weeks, including nuchal translucency, first- and second-trimester biochemical markers, anatomical scans, glucose challenge test, and the EFW before 32 gestational weeks. Also recorded were results of the definitive tests performed from week 32 onwards: advanced anatomical ultrasound scan, Doppler flow in various vessels (from diagnosis until delivery), amniotic fluid index, fetal echocardiography, genetic analyses (CMA, WES), infections and serology status (CMV, Toxoplasma), and coagulopathies (APLA, any other). Obstetric variables included the induction of labor, mode of delivery, GW at delivery, and birth weight/percentile. Neonatal parameters included gender, Apgar scores at 1 and 5 min, fetal arterial pH, admission to the neonatal intensive care unit (NICU) and neonatal morbidity, namely jaundice, transient tachypnea of the newborn (TTN), respiratory distress syndrome (RDS), sepsis, seizures, asphyxia, mechanical ventilation, necrotizing enterocolitis (NEC), intraventricular hemorrhage (IVH), hypoxic-ischemic encephalopathy (HIE), acidosis, meconium aspiration syndrome (MSA), and neonatal death.

### 2.5. Outcome Measures

The primary outcome was defined as a composite of neonatal outcomes. This was defined as at least one of the following: an Apgar score of less than 7 at 1 or 5 min, a fetal arterial pH of less than 7.1, admission to the NICU, jaundice, TTN, RDS, sepsis, seizures, asphyxia, mechanical ventilation, NEC, IVH, HIE, and death. Severe composite neonatal outcomes include asphyxia, HIE, IVH, meconium aspiration syndrome, seizures, intrauterine fetal death, and neonatal death.

### 2.6. Statistical Analysis

Statistical analysis was performed using SAS software, version 34.0 (SAS Corp. Cary, NC, USA). Descriptive statistics are presented by number and percentage for categorical variables and mean and standard deviation for continuous variables. Variables were compared between groups using chi-square test or one-way analysis of variance, as appropriate. Multivariate analysis was performed for the primary outcome, adjusting for maternal age, gestational week at delivery, and mode of delivery. The data are presented as an adjusted odds ratio (aOR) with 95% confidence interval (CI). A *p*-value below 0.05 was considered statistically significant.

### 2.7. Ethical Approval

The study was approved by the Institutional Review Board of Rabin Medical Center (approval no. 0727-17-RMC). Informed consent was waived by the Institutional Review Board due to the study’s retrospective design.

## 3. Results

Of the 791 women who gave birth to a newborn weighing below the 10th percentile (by gender and gestational age) during the study period, 79 were excluded because of maternal chronic diseases, 30 due to multifetal pregnancies, and 354 cases had missing data or were not followed in our clinic. This left 328 pregnancies for analysis. Of them, 77 (23.47%) with known late-onset FGR were diagnosed prenatally (known FGR, study group) and 251 (75.53%) were first diagnosed after birth (unknown FGR; control group). Their background and obstetric characteristics are shown in Table 1 and Table 2, respectively. The mean maternal age in the study group was 30.6 ± 5.2 years (range 19–46 years). There were no differences between the groups in rates of gestational diabetes (6.49% vs. 4.78%, *p* = 0.56) and hypertensive disorders of pregnancy (5.19% vs. 2.79%, *p* = 0.29). The unknown-FGR group had a significantly higher rate of previous CD (14.74% vs. 5.19%, *p* = 0.02) and Caucasians (100% vs. 92%, *p* = 0.04). The known-FGR group had higher rates of current CD (no trial of labor, 15.58% vs. 9.96%, *p* < 0.01) and labor induction (67.53% vs. 21.51%, *p* < 0.01). The other differences between the groups were not statistically significant.

The neonatal outcomes are shown in Table 3. The known-FGR group had a lower mean GW at delivery (37.6 ± 1 vs. 39.3 ± 1, *p* < 0.01), a higher rate of preterm deliveries prior to 37 gestational weeks (18.18% vs. 3.98%, *p* < 0.01), and a lower mean birthweight (2232 ± 292 vs. 2500 ± 200 g, *p* < 0.01).

The composite neonatal outcome was positive in 69 of the 328 neonates (21.03%). The rate was significantly higher in the known-FGR group than in the unknown-FGR group (38.96% vs. 15.53%, respectively; *p* = 0.01). The rate of severe composite neonatal outcome was also significantly higher in the known-FGR group (6.49% vs. 2.78%, respectively; *p* = 0.04). Isolated neonatal complications were also more frequent in the known-FGR group, but the differences did not reach statistical significance: sepsis (10.39% vs. 3.19%), TTN (2.60% vs. 0.4%), IVH (2.60% vs. 0), and NICU admission (7.79% vs. 1.99%). In the unknown-FGR group, there were cases of neonatal asphyxia (1.2%), need for mechanical ventilation (0.8%), meconium aspiration (1.2%), and an Apgar score less than or equal to 7 at 5 min (1.2%); none of these complications were found in the known-FGR group.

There was a single case of intrauterine fetal death in the unknown-FGR group, at 38 gestational weeks, following an uneventful pregnancy course. Two weeks earlier, a normal BPP had been recorded. The EFW at 32 gestational weeks was normal (25th percentile), with an actual birthweight below the third percentile. There were no cases of intrauterine death in the known-FGR group.

Within the known-FGR group, there was an etiological explanation for the FGR in only 22 cases (28.47%, Table 4). In the remainder (71.43%), the cause of the FGR was not determined. The most common etiology was placental insufficiency, in 15 cases (21.74%). Four women (5.19%) had, for the first time, an abnormal anatomical scan in late pregnancy, showing a small kidney, cardiomegaly, a dilated third ventricle, and a dilated renal pelvis. Three women (3.89%) tested positive for APLA, and one was diagnosed with fetal toxoplasmosis infection. The positive composite neonatal outcome rate was 45.45% in the FGR subgroup with an identified etiology and 36.6% in the FGR subgroup in which no etiology was identified (*p* = 0.6). The difference between each subgroup in the known-FGR group versus the entire unknown-FGR group was statistically significant (*p* = 0.01 and *p* = 0.01, respectively, Table 4).

Multivariate analysis, adjusted for maternal age, gestational age at delivery, and mode of delivery, yielded no differences between the known- and unknown-FGR groups for the composite neonatal outcome (aOR 1.73, CI 0.89–3.35, *p* = 0.1). Each additional week of gestational age at delivery was found to be protective (aOR = 0.7, 95% CI 0.56–0.86, *p* < 0.01).

## 4. Discussion

In this study, we sought to determine if the diagnosis of late-onset FGR and its corresponding workup and management during pregnancy affects neonatal outcomes. Our primary findings in a cohort of 328 neonates showed that the immediate outcomes were less favorable in those diagnosed with late-onset FGR during pregnancy as compared to those first diagnosed at birth. These included a lower gestational age of approximately 1.5 weeks at delivery, resulting in a 4.5-fold preterm birth rate. The intervention rate was significantly higher in this group as well. Most of the FGRs diagnosed during pregnancy were probably constitutional. When the cause was identified, it was most commonly uteroplacental insufficiency. On a multivariate regression analysis adjusted for maternal age, gestational age at delivery, and mode of delivery, there was no difference in the primary outcome between the groups.

Our findings are supported by several studies linking higher rates of obstetric complications in FGR pregnancies to the higher rate of interventions taken, such as induction of labor and elective cesarean delivery, resulting in an earlier gestational age at delivery and a higher likelihood of preterm birth and lower birthweight, both absolute and relative to gestational age [11,12,13]. Ohel et al. [12] found that the mean gestational age at delivery in patients diagnosed with FGR during pregnancy was 38.8 weeks versus 39.4 weeks in patients diagnosed only at birth. Corresponding values in the study of Nohuz et al. [16] were 37.7 and 39.4 weeks. Similarly, in our study, the gestational age at delivery of fetuses diagnosed with late-onset FGR during pregnancy was 1.5 weeks lower than that of fetuses diagnosed at birth, corresponding to a 4.5-fold preterm delivery rate. A more advanced gestational age at birth was found on multivariate analysis to be an independent protective factor for adverse neonatal outcomes. The neonatal composite outcome was statistically higher in the known-FGR group, regardless of whether the etiology was identified or not (Table 4). The main difference between the groups was the gestational week at delivery. However, due to the small size of the groups, the multivariate analysis was conducted between the two groups rather than three.

There is no consensus on the timing of delivery in late-onset FGR because of the lack of randomized trials based on Doppler indices. Guidelines for the management of FGR are highly variable [17], as the timing and route of delivery of FGR pregnancies are based on a combination of factors, including findings on Doppler, BPP, and NST coupled with the gestational age and EFW. The ISOUG [2] and ACOG [7] guidelines recommend that in FGR pregnancies with a normal Doppler study and a reassuring NST, delivery after 38 + 0 gestational weeks may be considered, but should not be delayed beyond 39 + 0 weeks to reduce the risk of severe growth restriction or stillbirth. In the only randomized control trial (DIGITAT), which included 650 patients with FGR, the randomly assigned cutoff for induction of labor or expectant monitoring was 36 gestational weeks [18]. The induction group gave birth 10 days earlier and had a neonatal birthweight of 130 g less than the expectantly managed group, but contrary to our results, the composite neonatal outcome rates were similar (6.1 and 5.3%) as were the cesarean delivery rates (approximately 14%). However, similar to our results, the study found that NICU admission was less likely when FGR fetuses were delivered after 38 GWs [19], suggesting a benefit of deferring delivery as long as the fetus is closely monitored and there are no other indications for early delivery. The authors of the DIGITAT study concluded that in cases of late-onset FGR, patients who are keen on non-intervention can safely choose expectant management and that it is reasonable to opt for labor induction to prevent possible neonatal morbidity and stillbirth.

In our study, among all the fetuses diagnosed with late-onset FGR during pregnancy, 38.96% had a positive composite neonatal outcome compared to 15.53% of the fetuses diagnosed with growth restriction at birth. Some previous studies support our findings whereas others contradict them [9,10,11,19,20]. The variations among the studies, including the DIGITAT trial [18,19], can be explained by different definitions and inconsistent neonatal outcome measures, as well as the lack of distinction between late- and early-onset FGR. According to some reports, neonates diagnosed with FGR during pregnancy had a significantly better immediate outcome, which they attributed to better management of pregnancy and delivery than cases of FGR diagnosed at birth [9,10,11]. However, not only did these studies fail to differentiate between late- and early-onset FGR, but their neonatal outcome measures were also different from those examined here. For instance, Verlijsdonk et al. [9] evaluated the primary outcomes of intrauterine death: a 5 min Apgar score less than 7, an umbilical artery pH less than 7.05, and a secondary outcome of NICU admission. Fratelli et al. [11] evaluated clinical and perinatal characteristics and found that identifying fetuses with growth delay during pregnancy could improve perinatal outcomes. Yet, they also found NICU admission to be more common in the group diagnosed with FGR during pregnancy, which was likely due to more frequent monitoring and higher rates of interventions in these cases, as well as more severe growth restriction. In our research, as in the DIGITAT trial, NICU admission was considered part of the composite outcome, and the rate was significantly higher in the known-FGR as compared to the unknown-FGR group. We have also distinguished between severe composite neonatal outcome and total adverse neonatal outcomes, with both being more prevalent in the study group.

A comparison of the mode of delivery between the groups revealed that most deliveries in the known-FGR group were initiated by induction and most in the unknown-FGR group were spontaneous. Additionally, the rate of cesarean delivery was lower in the unknown-FGR group, although the difference was not significant. Thus, when FGR is unknown, there seems to be a lower likelihood of any intervention, especially cesarean delivery. Despite the lack of statistical significance, these findings support previous studies showing higher rates of induction of labor and cesarean delivery in pregnancies with known FGR [9,10,11]. In the DIGITAT trial [19], the rate of cesarean delivery was similar in the two groups.

The prenatally diagnosed group in our study was further divided by etiology (identified or not identified), and each subgroup was compared with the unknown-FGR group for neonatal outcomes. The subgroup in which the etiology was identified had the highest composite neonatal morbidity rate, followed, in order, by the subgroup with unknown etiology and the control group. These findings are consistent with previous reports suggesting that fetuses with growth restriction due to a pathological cause have a higher rate of morbidity and mortality than constitutionally small fetuses [14,15,19,20], possibly because of the high incidence of early delivery and its consequences. They highlight the importance of accurate diagnosis, identification of the etiology, and categorization of fetuses into early- and late-onset groups as a primary measure to avoid unnecessary interventions and early delivery.

When growth restriction is diagnosed during pregnancy, especially if it is placenta-related, more frequent fetal monitoring is mandated. This is a double-edged sword. On one hand, it might lead to early intervention to prevent stillbirth, and on the other hand, it may be associated with a higher rate of preterm birth and adverse neonatal outcomes mainly due to complications of early-term delivery.

The strengths of our study include the specific definition of FGR, the relatively large number of women and infants, careful patient selection to avoid biases of background diseases and/or other complications prior to the time of diagnosis, and uniform follow-up and management policies under the same protocols of a single tertiary center.

The main limitation of our study is the retrospective design. Additionally, not all women in the study group underwent all possible diagnostic options (Doppler examination was performed on 86.61%, CMV serology test on 68.83%, toxoplasma serology test on 61.0%, fetal echo on 16.88%, and amniocentesis for genetic testing on 18.18%). This could have confounded the results, as women in whom there was a higher chance of finding a specific cause for FGR were referred for relevant diagnostic tests for that specific cause while other diagnostic tests were not performed, even though a complete workup was offered and is free. Moreover, due to the small size of the groups, the multivariate analysis was conducted between the known- and unknown-FGR groups, rather than between all three groups. In addition, long-term neonatal outcomes following hospital discharge were beyond the scope of the study. In addition, pregnancies that were not followed up in our clinics were excluded in order to use only valid in-house data.

## 5. Conclusions

A prenatal diagnosis of late FGR is associated with higher intervention and preterm birth rates compared to a diagnosis made after birth. This study emphasizes the importance of identifying fetuses with late-onset FGR during pregnancy and performing a specific and personalized assessment for each, to determine the cause and severity of the growth delay. Utilizing a combination of third-trimester anatomical surveys, genetic investigations, and Doppler criteria correlates with the likelihood of adverse perinatal outcomes in cases of late-onset FGR. In addition, it highlights the importance of caution when deciding to induce labor in cases of late-onset FGR in an attempt to avoid preterm delivery while maintaining close fetal monitoring.

## Figures and Tables

**Table 1:** Clinical characteristics of women with FGR pregnancies diagnosed prenatally (known FGR) or postnatally (unknown FGR).

Characteristics	Control Group Unknown FGR (n = 251)	Study Group Known FGR (n = 77)	*p*-Value
Age, years	30.55 ± 5.56	30.69 ± 5.26	0.74
Pre-pregnancy weight, Kg	56.96 ± 11.11	57.17 ± 12.07	0.92
Weight at birth, Kg	67.92 ± 10.98	67.57 ± 12.67	0.87
Gestational weight gain, Kg	10.79 ± 4.31	9.52 ± 3.68	0.13
Height, cm	160.01 ± 5.84	161.34 ± 6.99	0.25
Body mass index, Kg/m^2^	19.56 ± 8.06	21.04 ± 7.10	0.32
Gravidity	2 (1–3)	2 (1–3)	0.43
Parity	1 (0–2)	1 (0–2)	0.4
Previous cesarean delivery	37 (14.74)	4 (5.19)	0.02
Ethnicity			0.04
Caucasian	231 (92.03)	77 (100)	
Non-Caucasian	13 (5.18)	0 (0)	
Smoking during pregnancy	0 (0)	0 (0)	-
Alcohol consumption during pregnancy	0 (0)	0 (0)	-
Illicit drug use during pregnancy	0 (0)	0 (0)	-
Spontaneous conception	193 (76.89)	67 (87.01)	0.30

Data are presented as mean ± standard deviation for continuous variables or n (%) or median (range) for categorical variables.

**Table 2:** Obstetric characteristics of FGR pregnancies diagnosed prenatally (known FGR) or postnatally (unknown FGR).

Characteristics	Control Group Unknown FGR (n = 251)	Study Group Known FGR (n = 77)	*p*-Value
Cholestasis of pregnancy	0 (0)	0 (0)	
Gestational diabetes	12 (4.78)	5 (6.49)	0.56
Hypertensive disorders during pregnancy	7 (2.79)	4 (5.19)	0.29
Onset of labor			
Planned Cesarean delivery, no trial of labor	25 (9.96)	12 (15.58)	<0.01
Induction of labor	54 (21.51)	52 (67.53)	
Spontaneous	172 (68.53)	13 (16.88)	
Mode of delivery			
Vaginal	192 (76.49)	56 (72.73)	0.11
Vacuum-assisted	28 (11.16)	5 (6.49)	
Cesarean	31 (12.35)	16 (20.78)	
Type of cesarean			
Elective	21 (8.37)	11 (14.29)	1.0
Intrapartum	10 (3.98)	5 (6.49)	

Data are presented as n (%) for categorical variables.

**Table 3:** Neonatal outcomes of FGR pregnancies diagnosed prenatally (known FGR) or postnatally (unknown FGR).

Neonatal Outcomes	Control Group Unknown FGR (n = 251)	Study Group Known FGR (n = 77)	*p*-Value
Gestational age at birth, week	39.3 ± 1	37.6 ± 1	<0.01
Preterm birth (<37 + 0 weeks)	10 (3.98)	14 (18.18)	0.01
Birthweight, grams	2500.34 ± 200.87	2232.83 ± 292.05	<0.01
Head circumference, cm	34.63 ± 23.81	32.08 ± 1.07	0.19
Neonatal gender, male	121 (48.21)	41 (53.25)	0.51
Umbilical cord pH	7.32 ± 0.07	7.33 ± 0.05	0.39
Umbilical cord pH < 7.1	11 (4.38)	1 (1.30)	0.3
1 min Apgar score ≤ 7	8 (3.19)	0 (0)	0.2
5 min Apgar score ≤ 7	3 (1.20)	0 (0)	1.0
Asphyxia	3 (1.20)	0 (0)	1.0
Seizure	0 (0)	2 (2.60)	0.05
Hypoxic-ischemic encephalopathy	0 (0)	0 (0)	-
Intraventricular hemorrhage	0 (0)	2 (2.60)	0.05
Jaundice	21 (8.37)	17 (22.08)	0.02
Transient tachypnea of the newborn	1 (0.40)	2 (2.60)	0.13
Sepsis	8 (3.19)	8 (10.39)	0.02
Mechanical ventilation	2 (0.80)	0 (0)	1.0
NICU admission	5 (1.99)	6 (7.79)	0.02
Meconium aspiration syndrome	3 (1.20)	0 (0)	1.0
Respiratory distress syndrome	0 (0)	0 (0)	-
Necrotizing enterocolitis	0 (0)	0 (0)	-
Intrauterine fetal death	1 (0.40)	0 (0)	1.0
Neonatal death	0 (0)	0 (0)	-
Composite neonatal outcome *	39 (15.53)	30 (38.96)	0.01
Severe composite neonatal outcome **	7 (2.78)	5 (6.49)	0.04

Data are presented as mean ± standard deviation for continuous variable or n (%) for categorical variables. * Composite neonatal outcome includes all the above. ** Severe composite neonatal outcome includes asphyxia, hypoxic–ischemic encephalopathy, intraventricular hemorrhage, meconium aspiration syndrome, intrauterine fetal death and neonatal death.

**Table 4:** Obstetric and neonatal outcomes in pregnancies with known and unknown FGR, stratified by FGR etiology (identified, not identified).

Neonatal Outcomes	Control Group Unknown FGR (N = 251)	Known FGR		*p*-Value
		Etiology Identified (N = 55)	Etiology Not Identified (N = 22)	
Birthweight, grams	2500 ± 200	2249 ± 290	2192 ± 297	0.29 ^b^
Umbilical cord pH < 7.1	11 (4.38)	1 (1.82)	0 (0)	0.7 ^a^ 0.6 ^b^ 1.0 ^c^
1 min Apgar score ≤ 7	8 (3.19)	0 (0)	0 (0)	0.35 ^a^ 1.0 ^b^ 1.0 ^c^
5 min Apgar score ≤ 7	3 (1.20%)	0 (0)	0 (0)	1.0 ^a,b^
Asphyxia	3 (1.20)	0 (0)	0 (0)	1.0 ^a,b^
Seizure	0 (0)	2 (3.64)	0 (0)	0.03 ^a^ 1.0 ^c^
Hypoxic ischemic encephalopathy	0 (0)	0 (0)	0 (0)	-
Intraventricular hemorrhage	0 (0)	1 (1.82)	1 (4.55)	0.18 ^a^ 0.08 ^b^ 0.49 ^c^
Jaundice	21 (8.37)	11 (20)	6 (27.27)	0.02 ^a^ 0.01 ^b^ 0.54 ^c^
Transient tachypnea of the newborn	1 (0.40)	1 (1.82)	1 (4.55)	0.32 ^a^ 0.15 ^b^ 0.49 ^c^
Sepsis	8 (3.19)	7 (12.73)	1 (4.55)	0.08 ^a^ 0.53 ^b^ 0.42 ^c^
Mechanical ventilation	2 (0.80)	0 (0)	0 (0)	1.0 ^a b^
NICU admission	5 (1.99)	3 (5.45)	3 (13.64)	0.16 ^a^ 0.02 ^b^ 0.34 ^c^
Meconium aspiration syndrome	3 (1.20)	0 (0)	0 (0)	1.0 ^a^
Respiratory distress syndrome	0 (0)	0 (0)	0 (0)	-
Necrotizing enterocolitis	0 (0)	0 (0)	0 (0)	-
Intrauterine fetal death	1(0.4)	0 (0)	0 (0)	-
Neonatal death	0 (0)	0 (0)	0 (0)	-
Composite outcome *	39 (15.53)	20 (36.36)	10 (45.45)	0.01 ^a^ 0.01 ^b^ 0.6 ^c^

Data are presented as mean ± standard deviation for continuous variables or n (%) for categorical variables. ^a^ Comparison of unknown-FGR group with subgroup of known FGR of unidentified etiology; ^b^ comparison of unknown-FGR group with subgroup of known FGR with an identified etiology; ^c^ comparison of unknown-FGR group with the entire known-FGR group. * Composite neonatal outcome includes all the above.

## Data Availability

The datasets generated and analyzed during the current study are not publicly available due to ethical committee regulations but are available from the corresponding author on reasonable request.

## Abbreviations

BPP, biophysical profile; CMA, chromosome microarray analysis; CMV, cytomegalovirus; EFW, estimated fetal weight; FGR, fetal growth restriction; HIE, hypoxic–ischemic encephalopathy; IVH, intraventricular hemorrhage; MCA, middle cerebral artery; MSA, meconium aspiration syndrome; NEC, necrotizing enterocolitis; NICU, neonatal intensive care unit; NST, non-stress test; RDS, respiratory distress syndrome; TTN, transient tachypnea of the newborn; UA, uterine artery; WES, whole exome sequencing.
